# Subtle alterations of vestibulomotor functioning in conductive hearing loss

**DOI:** 10.3389/fnins.2023.1057551

**Published:** 2023-08-29

**Authors:** Francis A. M. Manno, Pikting Cheung, Vardhan Basnet, Muhammad Shehzad Khan, Yuqi Mao, Leilei Pan, Victor Ma, William C. Cho, Shile Tian, Ziqi An, Yanqiu Feng, Yi-Ling Cai, Martin Pienkowski, Condon Lau

**Affiliations:** ^1^Department of Physics, East Carolina University, Greenville, NC, United States; ^2^Department of Biomedical Engineering, Center for Imaging Science, Whiting School of Engineering, Johns Hopkins University, Baltimore, MD, United States; ^3^Center for Advanced Nuclear Safety and Sustainable Development, City University of Hong Kong, Kowloon, Hong Kong SAR, China; ^4^Department of Physics, City University of Hong Kong, Kowloon, Hong Kong SAR, China; ^5^Department of Nautical Injury Prevention, Faculty of Navy Medicine, Second Military Medical University, Shanghai, China; ^6^Department of Clinical Oncology, Queen Elizabeth Hospital, Kowloon, Hong Kong SAR, China; ^7^School of Biomedical Engineering, Southern Medical University, Guangzhou, China; ^8^Guangdong Provincial Key Laboratory of Medical Image Processing and Guangdong Province Engineering Laboratory for Medical Imaging and Diagnostic Technology, Southern Medical University, Guangzhou, China; ^9^Key Laboratory of Mental Health of the Ministry of Education, Guangdong-Hong Kong-Macao Greater Bay Area Center for Brain Science and Brain-Inspired Intelligence, Guangdong Province Key Laboratory of Psychiatric Disorders, Department of Neurobiology, School of Basic Medical Sciences, Southern Medical University, Guangzhou, China; ^10^Osborne College of Audiology, Salus University, Elkins Park, PA, United States

**Keywords:** vestibular dysfunction, eighth cranial nerve, vestibulocochlear, vestibular, motion, psychomotor

## Abstract

**Introduction:**

Conductive hearing loss (CHL) attenuates the ability to transmit air conducted sounds to the ear. In humans, severe hearing loss is often accompanied by alterations to other neural systems, such as the vestibular system; however, the inter-relations are not well understood. The overall goal of this study was to assess vestibular-related functioning proxies in a rat CHL model.

**Methods:**

Male Sprague–Dawley rats (*N*=134, 250g, 2months old) were used in a CHL model which produced a >20dB threshold shift induced by tympanic membrane puncture. Auditory brainstem response (ABRs) recordings were used to determine threshold depth at different times before and after CHL. ABR threshold depths were assessed both manually and by an automated ABR machine learning algorithm. Vestibular-related functioning proxy assessment was performed using the rotarod, balance beam, elevator vertical motion (EVM) and Ferris-wheel rotation (FWR) assays.

**Results:**

The Pre-CHL (control) threshold depth was 27.92dB±11.58dB compared to the Post-CHL threshold depth of 50.69dB±13.98dB (mean±SD) across the frequencies tested. The automated ABR machine learning algorithm determined the following threshold depths: Pre-CHL=24.3dB, Post-CHL same day=56dB, Post-CHL 7 days=41.16dB, and Post-CHL 1 month=32.5dB across the frequencies assessed (1, 2, 4, 8, 16, and 32kHz). Rotarod assessment of motor function was not significantly different between pre and post-CHL (~1week) rats for time duration (sec) or speed (RPM), albeit the former had a small effect size difference. Balance beam time to transverse was significantly longer for post-CHL rats, likely indicating a change in motor coordination. Further, failure to cross was only noted for CHL rats. The defection count was significantly reduced for CHL rats compared to control rats following FWR, but not EVM. The total distance traveled during open-field examination after EVM was significantly different between control and CHL rats, but not for FWR. The EVM is associated with linear acceleration (acting in the vertical plane: up-down) stimulating the saccule, while the FWR is associated with angular acceleration (centrifugal rotation about a circular axis) stimulating both otolith organs and semicircular canals; therefore, the difference in results could reflect the specific vestibular-organ functional role.

**Discussion:**

Less movement (EVM) and increase time to transverse (balance beam) may be associated with anxiety and alterations to defecation patterns (FWR) may result from autonomic disturbances due to the impact of hearing loss. In this regard, vestibulomotor deficits resulting in changes in balance and motion could be attributed to comodulation of auditory and vestibular functioning. Future studies should manipulate vestibular functioning directly in rats with CHL.

## 1. Introduction

### 1.1. Overview of conductive hearing loss

Conductive hearing loss (CHL) results in the attenuation of air conducted sound from the outer ear to the cochlea (Willcox and Artz, 2007). Albeit less severe than other forms of hearing loss, CHL is widespread. For example, a report indicating 19% of school aged children with some hearing loss, found 93% had CHL (Fitzpatrick et al., 2020). Further, 16.1% of the US population aged 20–69 had some form of unilateral and bilateral hearing loss (Agrawal et al., 2008). In the present study, rats were subjected to CHL to understand the effect on vestibular functioning due to hearing loss.

### 1.2. Relationship between conductive hearing loss and balance

Studies examining the relationship between CHL and balance have found mixed results. A review by Foster et al. (2022) found an inverse relationship between worsening postural stability and increased hearing impairment. Similarly, Horowitz et al. (2019) found a negative effect of CHL on balance. Studies have found an association between hearing loss and an increased risk of falling (Lin and Ferrucci, 2012). The correlation observed between falls and hearing loss may be explained by the concomitant dysfunction of the cochlea and vestibular sensory organ and limited access to auditory cues due to reduced hearing sensitivity (Lin and Ferrucci, 2012), or a decrease in attentional and cognitive resources resulting from hearing loss (Lin et al., 2011). However, some studies have shown no direct relationship between CHL and balance (Purchase-Helzner et al., 2004; Baraky et al., 2020). There is a complex relationship between CHL and balance underscoring the need for further research.

### 1.3. Relationship of conductive hearing loss to vestibular functioning

To-date, little is known concerning the comorbidity of vestibular dysfunction in hearing loss (Willcox and Artz, 2007). The vestibular nerve and cochlear nerve share a common course in the internal auditory canal to the brainstem (i.e., 8th cranial nerve, vestibulocochlear nerve; Rasmussen, 1940; Schefter and Harner, 1986; Ozdoğmuş et al., 2004). In the medulla, the nerves project to their respective nuclei (i.e., cochlear nuclei and vestibular nuclei; Brodal, 1974, p. 329; Harrison and Howe, 1974, p. 283). Ascending (afferent) and descending (efferent) projections form a network of connections in the brainstem for vestibular and cochlear processing, of which corticothalamic feedback (top down) is an important modulatory input for both systems (Hitier et al., 2014; Ventre-Dominey, 2014; Terreros and Delano, 2015; Wijesinghe et al., 2015; Mathews et al., 2017). Interestingly, vestibular dysfunction in humans is often found as a comorbidity to hearing loss (Antoine et al., 2017; Yu and Li, 2018a,b). In rats, high intensity noise induced hearing loss has been shown to be associated with vestibular dysfunction (Sohmer et al., 1999; Stewart et al., 2016, 2018). The inter-relation between the vestibular and auditory systems is further bolstered by several genes known to concomitantly affect both systems (Matsumoto et al., 2011; Ushakov et al., 2013; Antoine et al., 2017). In humans, a meta-analysis found individuals with an increased risk for sudden sensorineural hearing loss with vertigo had vestibular damage (Yu and Li, 2018a,b). A review in humans has highlighted susceptibility of the vestibular system to noise-induced hearing loss (Stewart et al., 2020).

### 1.4. Relationship between conductive hearing loss and postural stability/integration

Postural balance requires the central integration of vestibular, somatosensory, and visual inputs, and hearing may contribute as well (Horowitz et al., 2019). Interestingly an association has been found between hearing loss and an increased risk of falling (Viljanen et al., 2009; Lopez et al., 2011; Lin and Ferrucci, 2012). Moreover, studies by Kanegaonkar et al. (2012), Lubetzky et al. (2020), Anton et al. (2021), and Basta et al. (2023) have shown the association between improved postural control and the presence of auditory information. The ability to use auditory cues for postural control is diminished with hearing loss, despite patients with hearing aids overcoming the deficit (Vitkovic et al., 2016). In this regard, a study of simulated conductive hearing loss in humans found an impairment in the dynamic posturography test battery after earplugging (i.e., temporary hearing loss; Horowitz et al., 2019). A recent review found mixed evidence concerning the effect of hearing loss to postural stability (Carpenter and Campos, 2020). The authors concluded that when other sensory information is available the impact of hearing loss to stability is minimum; but, deemed hearing important with comorbid cognitive impairment or an impending balance disturbance (Carpenter and Campos, 2020). Studies in humans and rats are accumulating evidence that hearing loss often causes comorbidities associated with other sensory systems, such as the vestibular system.

### 1.5. Relationship between conductive hearing loss and anxiety

The relationship between hearing loss and anxiety is a known comorbidity (Fellinger et al., 2007; Nachtegaal et al., 2009; Chung et al., 2014). In a study by Mehta et al. (2003), the likelihood of developing anxiety symptoms was higher for older adults with hearing impairments. According to population-based studies, people with hearing impairments have a higher prevalence and risk of anxiety related disorders (Gomaa et al., 2013; Shoham et al., 2018). Moreover, the research by Ma et al. (2022) found a positive association between anxiety scores and high frequency hearing loss in age-related hearing loss. Similarly, hearing loss was found to be related to a greater possibility of developing anxiety symptoms in older adults (Contrera et al., 2016). Nevertheless, a study found the effects of hearing loss on mental health were moderate among young and middle-aged people, but almost absent among elderly people (Tambs, 2004).

### 1.6. Relationship between conductive hearing loss and autonomic responses

Several researchers have explored the association between CHL and autonomic responses. For example, a study by Mackersie et al. (2015) demonstrated that those with hearing loss exhibit decreased heart rate variability (HRV), a marker of autonomic function, under difficult listening conditions compared to people with normal hearing. Interestingly, enhanced HRV and increased parasympathetic activity has been demonstrated after receiving acoustic therapy in individuals with tinnitus (Reinhart et al., 2021). The aforementioned results imply the positive effect of restoration to auditory input on autonomic responses.

### 1.7. Vestibular functioning phenotyping assays

The following experiments use a vestibular phenotyping battery assessing the autonomic and behavioral effects of passive motion (Manno et al., 2020). Vestibular integrity tests such as the rotarod and balance beam assess the presence of vestibular dysfunction by examining gross deficits (i.e., locomotion impairments). Additionally, vestibular stimulation tests were performed, such as the elevator vertical motion (linear acceleration) and the Ferris-wheel rotation (centrifugal rotation), to assess functioning of the saccule and otoliths/semicircular canals, respectively, for examining the responses to vertical and horizontal plane motion (i.e., passive motion challenge tests). The vestibular stimulation tests simulate motion sickness leading to an autonomic reaction, as these tests directly stimulate the otolith organs by linear acceleration and the semicircular canals by angular acceleration (Manno et al., 2020). Impairments in these assays could establish vestibular dysfunction on a gross level (i.e., falling off a balance beam) or potentially pertain to the saccule (i.e., elevator vertical motion) and otoliths/semicircular canal (i.e., Ferris-wheel rotation); (i.e., reduced motion after stimulation). In this regard, significant advances have been made by understanding the neural and behavioral aspects of motion sickness (Cai et al., 2007, 2010; Wang et al., 2012, 2015, 2017; Zhou et al., 2017; Qi et al., 2019; Manno et al., 2020), likely due to a sensory conflict or neuronal mismatch from receiving integrated motion information that differs from the anticipated internal model of the environment (Reason and Brand, 1975; Reason, 1978). The elevator vertical motion and the Ferris-wheel rotation use stimulation of the saccule and otoliths/semicircular canals as a proxy for vestibular dysfunction. Our group has made significant advances neuroimaging auditory functioning and hearing loss (Dong et al., 2018; Lau et al., 2018; Wong et al., 2020; Manno et al., 2021a,b); therefore, we were interested in whether hearing loss models encounter significant comorbidity with vestibular dysfunction(s). Considering this line of inquiry, we applied our vestibular phenotyping battery to test motion and balance (Manno et al., 2020) in a rat model of conductive hearing loss (Manno et al., 2019, 2021a).

The rotarod is one of the standard assessments of motor functioning (Hamm et al., 1994; Crabbe et al., 1999; Bohlen et al., 2009; Brooks et al., 2012) and can determine aspects of a motor strategy for rotarod skill learning (Buitrago et al., 2004; Shiotsuki et al., 2010). The rotarod is an examination of motor coordination (Fujimoto et al., 2004; Deacon, 2013) and a sensitive index of injury-induced motor dysfunction (Hamm et al., 1994). For our purpose, rotarod measures intact vestibular functioning (i.e., vestibular integrity) as several models have used the assay for assessing vestibular dysfunction (Isgrig et al., 2017; i.e., 3,3′-iminodipropionitrile: Schlecker et al., 2011; Negishi-Oshino et al., 2019; vestibulotoxicity using an intratympanic gentamicin injection: Zhai et al., 2019; Kim et al., 2021; cochleostomy: Suh et al., 2016: labyrinthectomy: Chang et al., 2018). Interestingly, chronic exposure to low frequency noise (below 0.5 kHz) at moderate levels of 60–70 dB has been shown to induce balance impairments as assessed by rotarod performance retention time (Tamura et al., 2012).

The balance beam assay for mice (Luong et al., 2011) and rats (Modianos and Pfaff, 1976; Feeney et al., 1982; Goldstein and Davis, 1990; Piot-Grosjean et al., 2001; Kalueff et al., 2005; Sweis et al., 2016) can be used to study anxiety (Kalueff et al., 2005; Sweis et al., 2016), and traumatic injury (Goldstein and Davis, 1990; Piot-Grosjean et al., 2001; Kline et al., 2007; Cheng et al., 2012; Sweis et al., 2016; Hausser et al., 2018; Weeks et al., 2018). The balance beam is an examination of gross vestibulomotor integrity (Hausser et al., 2018), fine motor coordination (Luong et al., 2011), and locomotion (Feeney et al., 1982) and can be used as a sensitive index of behavioral deficits such as posture and movement dysfunction (Modianos and Pfaff, 1976). For our purpose, balance beam measures vestibular integrity as several models have used the assay for assessing locomotion-related vestibular dysfunction (i.e., motion sickness; Zhou et al., 2017; Qi et al., 2019; Zhong et al., 2022; chronic exposure to 16.4 T ultra-high B_0_ magnetic fields: Tkáċ et al., 2021; knockout loss of function models: Takimoto et al., 2018; Ono et al., 2020; Chang et al., 2022; pharmacological challenge: Li et al., 2017; Han et al., 2021). Interestingly, deficits in motor coordination as revealed by the balance beam have been used to assess children with hearing-impairments, forming a component of the Bruininks-Oseretsky test of motor proficiency (Wiegersma and Van der Velde, 1983; Dummer et al., 1996; De Kegel et al., 2010, 2012; Maes et al., 2014).

The elevator vertical motion and Ferris-wheel rotation tests are assays of vestibular stimulation using passive motion, coupled with outcome metrics in locomotion and defecation (the latter a proxy for autonomic dysfunction as rodents have no emetic reflex, i.e., they do not vomit: Brading and Ramalingam, 2006; Horn et al., 2013). The background here is passive motion stimulating the otolith organs, the saccule by linear accelerations in the vertical plane (up-down) and the utricle by linear accelerations in the horizontal plane (forward-backward; Büttner-Ennever, 1999) and the semicircular canals (i.e., anterior, posterior, and horizontal) by angular acceleration (Riccio and Thach, 1968; Armstrong et al., 2015; Rabbitt, 2019) may result in impairments to sensing motion, thereby locomotion deficits, or an autonomic reaction as in motion sickness. In that regard, these assays could alter processes such as defecation (used as a proxy for autonomic reaction in motion sickness: Manno et al., 2020). The elevator vertical motion (up-down: similar to the pitch and roll of a ship encountering a wave), assesses vestibular functioning by stimulating the otolith sensory organs which encode linear accelerations (i.e., the saccule responds to movements in the vertical plane; Büttner-Ennever, 1999). The Ferris-wheel rotation (centrifugal rotation about a circular axis: similar to whirling in a circle around a baseball bat) device stimulates the otolith organs by linear acceleration and the semicircular canals by angular acceleration (Riccio et al., 1968; Armstrong et al., 2015). We developed these tests and the outcome metrics as behavioral proxies for vestibulomotor dysfunction in motion sickness. Both devices induce vestibular stimulation leading to symptoms of autonomic reaction (mimicking motion sickness in rodents, i.e., causing defecation) and alterations to locomotion behavior (Manno et al., 2020). These assays for motion sickness have a link to the vestibular system and therefore we decided to use them as proxies for determining vestibular dysfunction, which may occur in hearing loss.

### 1.8. Research questions

Due to the intimate relationship between auditory and vestibular system (Ryugo et al., 2011), in addition to the known comorbidity of hearing loss with vestibular dysfunction (Yu and Li, 2018a,b), we sought to determine if a rat model of conductive hearing loss would be accompanied by vestibular-related disorder(s). We used a battery of behavioral assays commonly used for evaluating motion sickness (Cai et al., 2007, 2010; Wang et al., 2012, 2015, 2017; Zhou et al., 2017; Qi et al., 2019; Manno et al., 2020). In that regard, disorders of the autonomic system in rats could manifest as symptoms (i.e., autonomic reaction) which are easy to differentiate and enumerate (i.e., epigastric effects—changes in defection: Balaban, 1999). The primary aim was to evaluate the CHL rodent model to determine if vestibular functioning was impaired. Here, we found balance and autonomic alterations (defecation proxy) resulting from CHL, despite previous research not reporting secondary effects accompanying conductive hearing loss in rodent models (Kotak et al., 2008; Liberman et al., 2015). Future studies should elucidate the mechanisms involved in hearing loss accompanied vestibular-related dysfunction by directly manipulating the vestibule.

## 2. Methods

### 2.1. Animal preparation

The present study and procedures were approved by the Committee on the Use of Live Animals in Teaching and Research at the City University of Hong Kong in accordance with the Guide for the Care and Use of Laboratory Animals (National Research Council, 1996). Male Sprague–Dawley rats (*n* = 134; 250 g), 2 months old, were examined for the present study. Rats were randomly assigned to groups. Rats were caged under a constant temperature (25°C) and 60 to 70% humidity. After CHL surgery, animals were returned to their home cages under a warm lamp in a heated room (35°C) for recovery. Rats were housed under a 12:12-h light/dark circadian cycle with *ad libitum* access to food and water. Rats were acclimated to the housing environment for at least 1 day prior to experiments in the behavioral battery or CHL. Anti-inflammatory drugs were supplied in the water for 1 week (Manno et al., 2019).

Each experiment consisted of naïve rats, used only once for each experiment to reduce stress caused by handling (Prager et al., 2011): auditory brainstem response (*n* = 12 over 4 periods: Pre-CHL, Post-CHL same day, Post-CHL 7 days, and Post-CHL 1 month) rotarod (*n* = 30), balance beam (*n* = 20), EVM (*n* = 36), and FWR (*n* = 36). Controls for EVM and FWR were naïve as the evaluation to a novel environment was required. For balance beam and rotarod assessments, rats were used as their self-control for all experiments and subsequently conductive hearing loss was induced.

### 2.2. Conductive hearing loss induction

**Figure 1:**
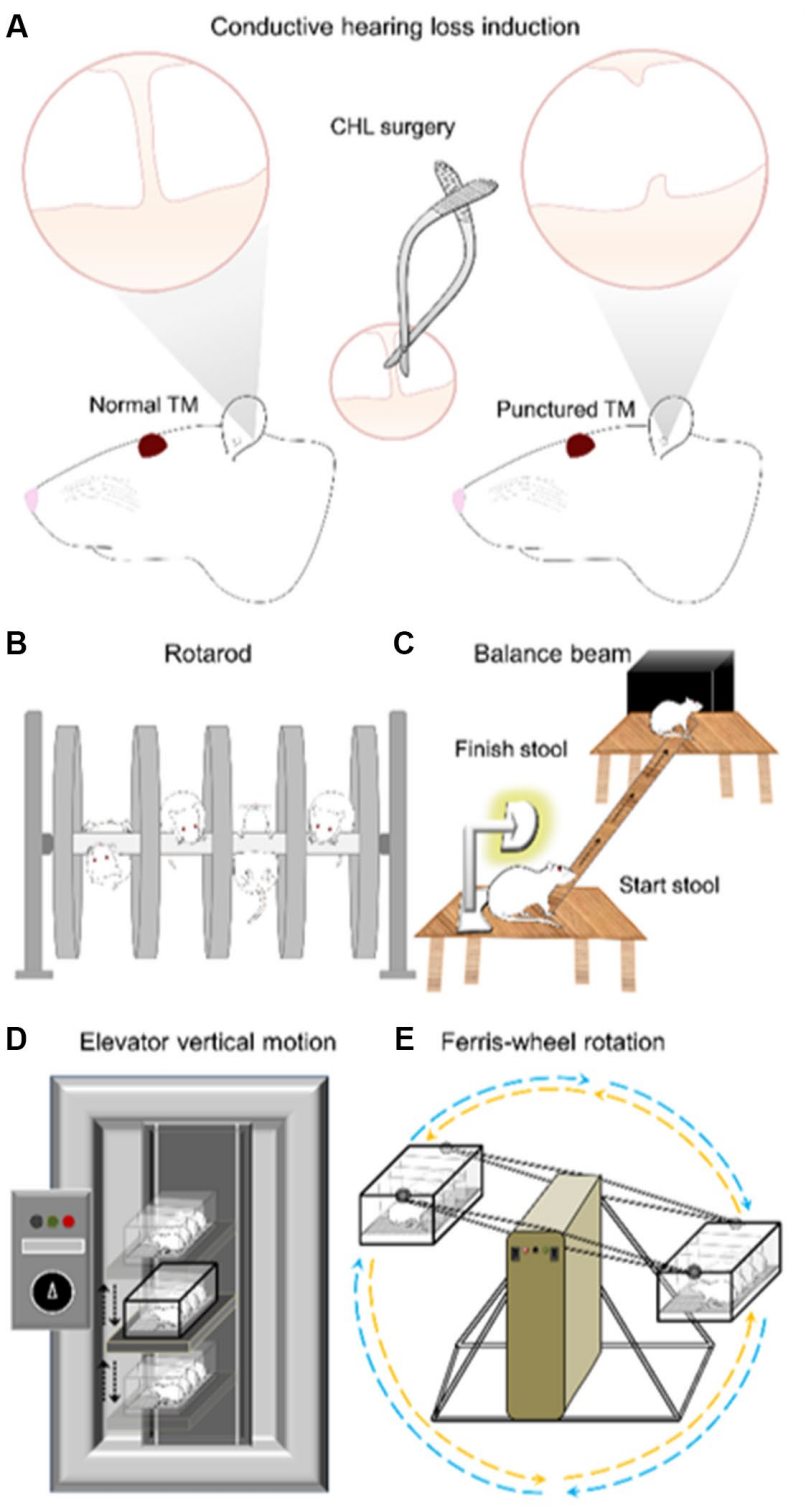
Experimental diagram of conductive hearing loss (CHL) surgery, rotarod, balance beam, elevator vertical motion and Ferris-wheel rotation device. **(A)** The CHL surgery was conducted by tympanic membrane (TM) puncture and malleus displacement (Manno et al., 2019). **(B)** Rotarod was conducted from 4 to 40 RPM with unlimited duration. Training was for 3 days followed by a 4th day evaluation. The CHL surgery was conducted after the last training session on the 4th day. The 5th, 6th, and 7th day were test periods of assessment. **(C)** Balance beam assessment was conducted during three training days promoting the rat to transverse the beam during 5 min periods. The 4th day was used as evaluation. CHL was induced after day-4 session. The comparison was day-4 (pre-CHL) and post-CHL day-5. **(D)** The elevator vertical motion device moves in a vertical fashion with an amplitude 22 cm up and 22 cm down from neutral (linear acceleration device in the vertical plane stimulating the saccule). The test session is 2.5 h. **(E)** The Ferris-wheel rotates in a clockwise-pause-counterclockwise cycle of ~10 s (centrifugal rotation device stimulating the otolith organs (saccule and utricle) by linear acceleration (up-down back-forth) and the semicircular canals by angular acceleration). The test session is 2 h. Elevator vertical motion and Ferris-wheel rotation devices are evaluated with defecation counting and open field examination.

**Figure 2:**
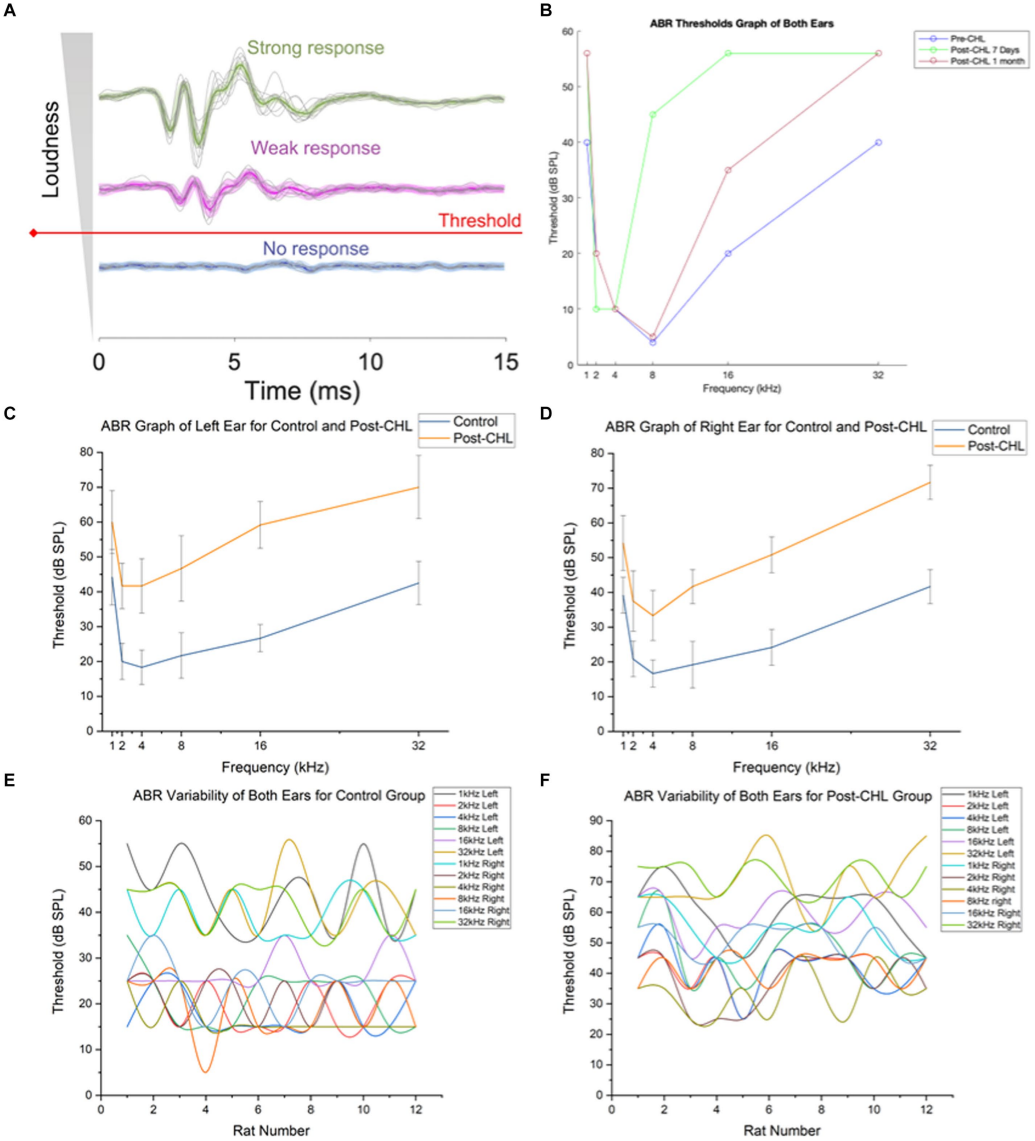
Auditory brainstem response and design for automated threshold determination. **(A)** Typical level representation of ABRs. The median ABR response is bolded in a color line. Obtaining a stable waveform necessitated level averaging of sweep recordings at a strong stimulus (green) compared to that of weak response (magenta), whereas level averaging did not improve the signal to noise ratio (SNR) of subthreshold recordings containing no response (blue). **(B)** The machine learning algorithm of ABR discerning threshold responses utilized both ears for Pre-CHL, Post-CHL 7 Days, and Post-CHL 1 Month. Note Post-CHL Same day is absent due to considerable noise and variability in ML thresholding procedure. Auditory brainstem responses (ABR) to tones recorded from **(C)** Left and **(D)** Right ears in control (orange) and 7 days after conductive hearing loss (CHL) induction, Post-CHL (blue). Averaged variability in ABR for **(E)** control and **(F)** for 7 days Post-CHL for both ears by individual rat. Note the shift in dB threshold upward.

The CHL induction was adapted from similar procedures (Figure 1A; Buran et al., 2014; Manno et al., 2019, 2021a). Rats were anaesthetized with a mixture of ketamine and xylazine (80–100 mg/kg: 5–10 mg/kg) administered *via* intraperitoneal injection. A surgical field was setup and the head of the rat was aligned along the prone position, closest to the surgeon. The helix of the rat ear was extended to cause the external auditory canal (i.e., ear canal, external auditory meatus) to be aligned perpendicular to the plane of the tympanic membrane (TM). Micro-scissors (Micro spring scissors, RWD Life Science, S11035-08) were introduced into the center of the auditory canal being careful not to damage the auditory canal (Figure 1A). The scissors were introduced slowly and proceeding a short distance, approximately 5 mm from the center of the obscurity. The scissors were then thrusted forward gently through the center of the TM. The thrusting elicited TM puncture. With the micro-scissors in the appropriate position and correct depth, a “pop” sound was audible when the micro-scissor tips punctured the TM displacing the malleus. The popping sound was approximately 20 dB sound pressure level (SPL) above background sound as recorded by a high frequency microphone (M50, Earthworks, Chesterfield, MO). After puncturing the TM, the micro-scissors were immediately opened and rotated three times to ensure displacement of the ossicles, primarily the head of the malleus. The micro-scissors were removed, and the rat placed under an otoscope to visualize and ensure TM puncture (Otoscope mini 3,000, HEINE, D-008.70.120 M, Standard LED otoscope). Each rat was evaluated to ensure no significant bleeding occurred after the surgical procedure. Further, each rat was evaluated before and after CHL underneath the otoscope for visualization and confirmation of a normal TM and a damaged TM after CHL induction. After CHL induction, the head of the malleus should be missing, and the TM punctured. The CHL procedure resulted in an >20 dB threshold shift as confirmed by auditory brainstem response after TM puncture (see Figure 2).

### 2.3. Auditory brainstem response methods

The auditory brainstem response (ABR) methods were adopted from mice (Pienkowski, 2018) using a Tucker Davis Technologies (TDT, Model MF1) setup measuring frequency response between 1 and 32 kHz before and after CHL. Stimuli were tone bursts at 1, 2, 4, 8, 16, and 32 kHz, 3 ms in duration including 1 ms cos2 on and off ramps. Stimuli were synthesized using TDT software (SigGen), converted to analog (TDT, RZ6), and played out by a TDT MF1 speaker coupled to the rats ear canal with a 10-cm tube and sealed probe (system flat to 20 dB from 1 to 32 kHz). Sound levels were calibrated with the probe coupled to a quarter-inch microphone (ACO Pacific, 7017) with an additional 7-mm-long plastic tube, intended to approximate the length of the ear canal. Stimuli were presented at 10–90 dB SPL at each frequency, in 10 dB steps, with 512 repetitions per level and a presentation rate of 21.1/s. The ABR was recorded differentially between the left mastoid area and vertex (ground electrode in the nape of the neck) using subdermal needle electrodes (Rochester Electro-Medical, Lutz, FL, United States, Model S83018-R9). Potentials were amplified 5,000× [TDT, RA4LI headstage (20×) and RA4PA pre-amp (250×)], digitized, and filtered between 100 and 3,000 Hz (TDT, RZ6), under the control of TDT software (BioSig, Alachua, FL, United States). At low stimulus levels, measurements were repeated twice, and ABR threshold was defined as the lowest SPL that yielded a reproducible ABR, minus 5 dB (half the step size). We performed manual ABR thresholding (M.P.) and automated ABR thresholding as described below.

ABR thresholds were quantitatively determined using a machine learning algorithm originally devised for mice (automated threshold determination; Wang et al., 2021). The datasets were arranged into a format including both ears. In total there were 70 datasets for Pre-CHL, 63 datasets for Post-CHL same day, and 27 datasets for both Post-CHL 7 days and Post-CHL 1 month. The decrease in number of datasets represents rats exiting the study. Each dataset was repeated and averaged individually by an iterated method. The modified part of this algorithm started with the highest stimulus level (60 dB SPL was used for threshold determination) and iteration count (244 counts in this experiment), followed by re-arranging a number of sweep batches (waveforms) cumulatively averaged in three data buffers (33 sweep batches for Pre-CHL, 14 sweep batches for Post-CHL same day, and 9 sweep batches for both Post-CHL 7 days and Post-CHL 1 month), 3 batches, 6 groups total for each. Each batch is averaged and stored in the mean group with left and right ears combined. Originally, if the absolute lag was less than the allowed value (L), the outcome returned a true with a suggested positive response. However in our case, the L was set to be L ≤ 1, because our data is more of a modified dataset as apposed to raw data in real time (Wang et al., 2021). The acquired ABR dataset had 10-dB step size and was fitted with both exponential and sigmoidal functions. Theoretically, the approximated stimulus level yielded 1.0–0.9 on the best-fitted exponential or sigmoidal function, and this was modified to be 0.99995 in this test. The thresholds were reported at Pre-CHL, Post-CHL same day, Post-CHL 7 days, and Post-CHL 1 month.

### 2.4. Behavioral battery

#### 2.4.1. Rotarod

The Rotarod test (Figure 1B; Rotamex-5, Columbus Instruments, Columbus Ohio: 0254-2002L) was conducted from 4 to 40 revolutions per minute (RPM; start to finish), with unlimited duration. The acceleration interval was 1 s and the acceleration step speed was 0.3 RPM with a counter clockwise rotation. The protocol implemented a 5 s help period in which rats were helped if they fell off within 5 s of beginning the training session. Two training sessions (5 min each) were conducted per day for the first 2 days. A 10 min training session was conducted on the third and fourth day, respectively. The CHL surgery was conducted on the fourth day after the 10 min training session. An evaluation was conducted on the 5th, 6th, and 7th day with two sessions per day. The evaluation had two parameters: speed (RPM) and time duration on the rotarod (sec). A sound meter was used to confirm the rotarod sound level (LINI-T UT353 BT Mini Sound Meter).

#### 2.4.2. Balance beam

The balance beam (Figure 1C) was setup by placing two wooden stools (each 0.75 cm in height) approximately 110 cm apart (Luong et al., 2011; Wang et al., 2017; Zhou et al., 2017; Qi et al., 2019; Manno et al., 2020). A lamp was placed at the start-stool and an opaque plastic box (15 cm × 15 cm × 8 cm) on the finish-stool. A narrow wooden beam (2.5 cm × 130 cm) was placed between the two stools allowing 100 cm distance between the stool edges, from start-stool to finish-stool (Figure 1C). The lights were turned off in the room during the experiment to ensure the rat followed the direction of the balance beam from lighted to darkened region. Rats were trained for 3 days, which consisted of daily acclimatization and accommodation of the rat to the balance beam. The training promoted the rat to cross (transverse) the beam during 5 min periods until eventually the rat would cross of its own volition successively. A generic stopwatch was used to time start to finish. Once a stable baseline start-to-finish balance beam crossing time was achieved after 3 days training, the 4th day was used as the evaluation. After the day-4 session, CHL was induced. The comparison was day-4 and post-CHL day 5. The before and after CHL measures were taken on consecutive days. The evaluation parameter was time to transverse (sec). If rats did not cross within a 5 min period during the evaluation phase, a designation “no movement” was given.

#### 2.4.3. Elevator vertical motion

The present experiment used a custom EVM device (Figure 1D) with a 22 cm up and 22 cm down vertical range of motion with a maximum cycle period of 1,000 ms (1 s). Four rats were placed in a Plexiglas box at a time and then subsequently, the box was secured to the elevator pad of the EVM device. The amplitude setting (up-down distance) was 22 cm up and 22 cm down from neutral. The vertical motion was set initially to 2,500 ms period for 5 min (i.e lowest setting for acclimatization), followed by 2000 ms for 5 min, and followed by 1,500 ms for 5 min. The test period was 1,000 ms period for 2 h. At the end, the device was slowed in reverse, set to 1,500 ms period or 5 min, followed by 2,000 ms for 5 min, and followed by 2,500 ms for 5 min. The total experimental time was 2.5 h, after which the EVM was stopped and the rats evaluated using defecation counting and open-field examination (Manno et al., 2020). CHL rats were evaluated 7 days after TM puncture using EVM. Comparisons were made to control rats.

#### 2.4.4. Ferris-wheel rotation

The FWR device (Figure 1E) was based on an early rotational stimulator for motion sickness induction (Crampton and Lucot, 1985). The FWR device is an angular acceleration and deceleration stimulator (Cai et al., 2007, 2010; Wang et al., 2012, 2015, 2017; Zhou et al., 2017; Qi et al., 2019; Manno et al., 2020). Rats were placed in a custom plexiglass container with the long axis of the body perpendicular to the horizontal rotation rod of the Ferris-wheel. This placement ensures stimulation of the otolith organs (utricle: anterior–posterior and saccule: vertical direction) during rotation. The FWR was started by rotating in a clockwise direction at 16 °/s^2^ to reach an angular velocity of 120 °/s and then decelerated at 48 °/s^2^ to reach 0 °/s. After a 1 s pause, the rotation was continued in a counter-clockwise manner similar to above (16 °/s^2^ to reach an angular velocity of 120 °/s and then decelerated at 48 °/s^2^ to reach 0 °/s; Cai et al., 2007, 2010; Wang et al., 2012, 2015, 2017; Zhou et al., 2017; Qi et al., 2019; Manno et al., 2020). The clockwise-pause-counterclockwise cycle lasted approximately 10 s, before returning to the initial position. The rotation period was 2 h per session and performed in darkness to minimize visual cues. The FWR was then stopped and rats were evaluated using defecation counting and open-field examination (Cai et al., 2007, 2010; Wang et al., 2012, 2015, 2017; Zhou et al., 2017; Qi et al., 2019; Manno et al., 2020). CHL rats were evaluated 7 days after TM puncture using FWR. Comparisons were made to control rats.

#### 2.4.5. Defecation counting

Defecation counting was performed after EVM and FWR to quantify autonomic effects of the passive motion. The protocol (Manno et al., 2020) enumerated ‘poop’ pellets (fecal boli). Rats were removed from the Plexiglas containers after EVM and FWR and fecal boli within the container attributed to the rat were enumerated. Complete and semi-complete ‘poop’ (fecal boli) were counted. A baseline ‘poop’ measurement was obtained for a 2.5-h period for comparative purposes attributed to each rat. The chronology involved enumerating ‘poop’ (fecal boli) pre-EVM/pre-FVM followed by EVM or FVM fecal boli counting performed during the aforementioned experiments.

#### 2.4.6. Open-field examination

Open-field examination was performed after EVM and FWR to quantify locomotion effects of the passive motion. The protocol (Manno et al., 2020) measured total distance traveled (in cm) in the open-field box. Open-field motion was recorded using an infrared video camera for 3 min after EVM and FWR (Gao et al., 2014; Lopes et al., 2015; Aitken et al., 2017; Manno et al., 2020). The rat was not placed in the open-field box before EVM or FWR since the environment must be novel and the animal naïve. The data herein was analyzed using Ethovision™ analysis of open-field behavior (Aitken et al., 2017), but several open-source software pipelines exist for behavioral video analysis such as Bonsai (Lopes et al., 2015) and Matlab implementations that use frame-by frame differences (Gao et al., 2014; Manno et al., 2020, 2022, 2023).

### 2.5. Statistical analysis

A one-way ANOVA and *t*-tests were used to determine differences between CHL and control groups with a significant P level set at 0.05 (Zar, 1999). Effect size statistics Cohen’s *d* and Pearson correlation coefficient effect size *r* (Cohen, 1992) were used for determining the effect of hearing loss. For Cohen’s *d* effect size was delineated by the following criterion small ≥0.2, medium ≥0.5, and large ≥0.8 effects (Cohen, 1992). For effect size, *r* effect size was delineated by the following criterion: small ≥0.1, medium ≥0.3, and large ≥0.5 effects (Cohen, 1992). Box and whisker plots were made to display the data visually (McGill et al., 1977; Tukey, 1977). The maximum and minimum are represented by error bars (i.e., whiskers) extending from the box (i.e., lower and upper hinges). Outliers (unbounded dots) are defined as datapoints below Q1–1.5 IQR or above Q3 + 1.5 IQR.

## 3. Results

### 3.1. Auditory brainstem response in conductive hearing loss

The ABR process utilized a manual and automated threshold determination using machine learning (Figure 2A). The ABR ML threshold procedure found an average dB for Pre-CHL = 22.3 dB, Post-CHL same day = 56 dB, Post-CHL 7 days = 38.83 dB, and Post-CHL 1 month = 30.33 dB across the frequencies assessed (1, 2, 4, 8, 16, and 32 kHz: Figure 2B). At 8 and 16 kHz (above and below the recovery), the Pre-CHL threshold was 4 dB and 20 dB respectively, with a large difference observed after the CHL procedure (Post-CHL 7 days, was 45 and 56 dB, at 8 and 16 kHz, respectively). Some recovery was observed at Post-CHL 1 month in the lower frequencies; for example, the average difference between the Pre and Post -CHL for 8 kHz red and blue data points was 4.25 dB, although above 16 kHz the average difference was 15.5 dB (Figure 2B).

Using the manual determination (M.P.), the ABR threshold difference was significant for the left ear (*F* = 31.23, df = 5, *p* = 6.42E-15; Figure 2C) and right ear (*F* = 64.33, df = 5, *p* = 9.68E-22; Figure 2D) post-CHL compared to the control (Pre-CHL). There was variability in both Pre-CHL (control) and post-CHL ABR individual rats’ response across the frequencies assessed (1, 2, 4, 8, 16, and 32 kHz; Figures 2E,F). The ABR procedures confirmed significant hearing loss from Pre-CHL (control) 27.92 dB ± 11.58 dB threshold compared to Post-CHL 50.69 dB ± 13.98 dB (mean ± SD) threshold across frequencies tested.

### 3.2. Rotarod

**Figure 3:**
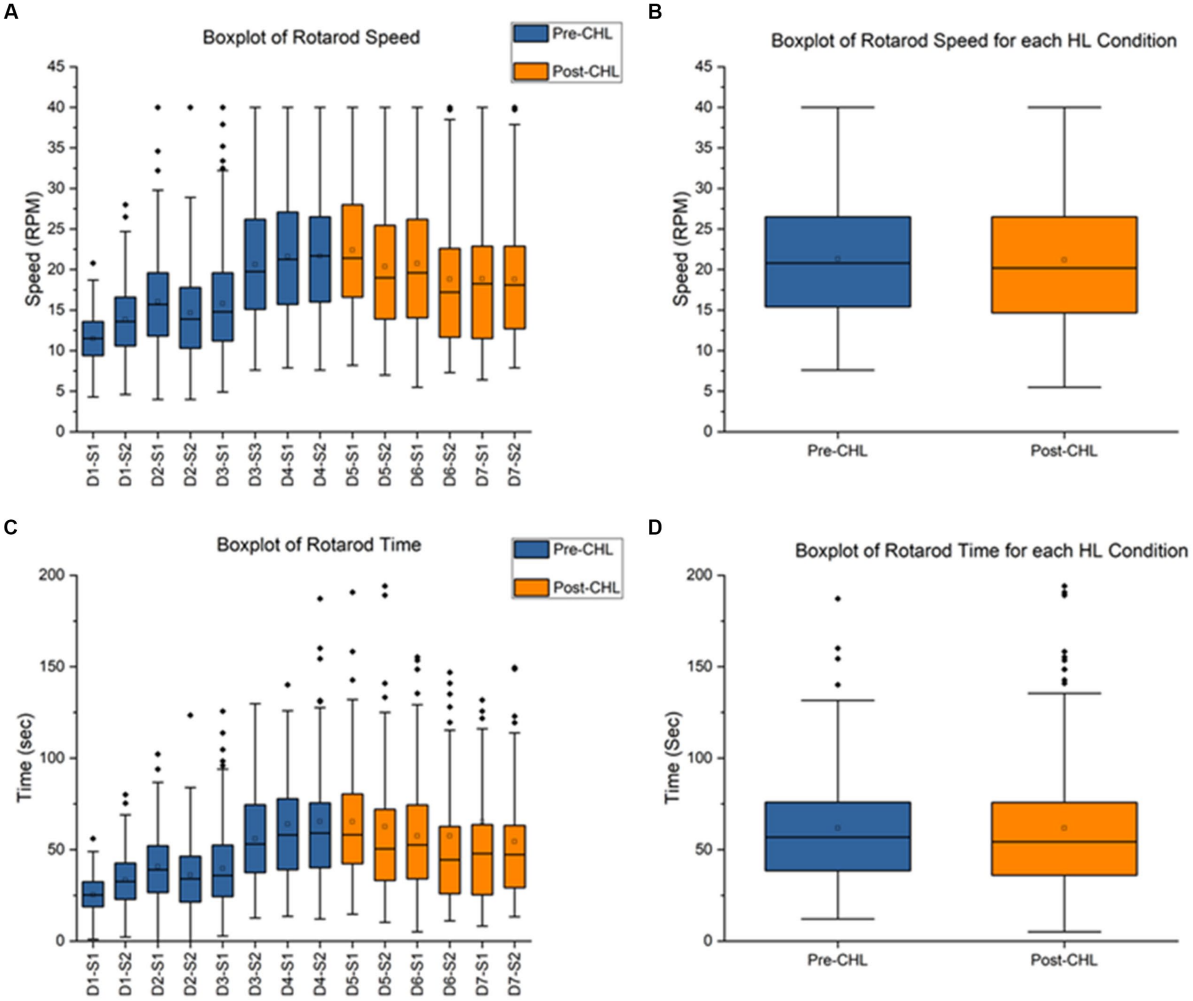
Rotarod pre-CHL and post-CHL for speed (RPM) and time duration (sec). Sample size of rotarod (*n* = 30). **(A)** Box and whisker plot of speed (RPM) for the series of test days and sessions. Pre-CHL is represented in blue and post-CHL is represented in orange. Abbreviations like D1-S1 for Day 1- Session 1 have been used to represent different days and sessions, respectively. All values were plotted. Note the considerable overlap between days before Day-4-Session-2 pre-CHL and days after Day-5-Session-1 post-CHL. The following results were obtained for a one-way ANOVA test between different days and sessions: *F* = 41.5, df = 13, *p* = 2E-98. **(B)** Box and whisker plot of speed (RPM) for pre and post-CHL. Pre-CHL is represented in blue and post-CHL is represented in orange. Note the considerable overlap. **(C)** Box and whisker plot of time (sec) for the series of days and test sessions. Pre-CHL is represented in blue and post-CHL is represented in orange. Abbreviations like D1-S1 for Day 1- Session 1 have been used to represent different days and sessions, respectively. Some outlier dots are not plotted. Note the considerable overlap between days before Day-4-Session-2 pre-CHL and days after Day-5-Session-1 post-CHL. The following results were obtained for a one-way ANOVA test between different days and sessions: *F* = 11.9, df = 13, *p* = 9.23E-26. **(D)** Box and whisker plot of time (sec) sessions pre-CHL and sessions post-CHL. Pre-CHL is represented in blue and post-CHL is represented in orange. Some outlier dots are not plotted. Note the considerable overlap.

The pre-CHL mean was 21.67 ± 8.13 RPM and post-CHL mean was 22.40 ± 7.76 RPM, indicating overlap in SD. A one-way ANOVA between different days and sessions for RPM was significantly different (*F* = 41.5, df = 13, *p* = 2E-98; Figure 3A). A paired *t*-test was not significant (*t*_239_ = 1.0916, *p* = 0.2761; Figure 3B). For speed RPM, Cohen’s *d* was 0.0919 and effect size *r* was 0.0459. The direction of change was an increase in speed for CHL. The pre-CHL mean was 65.39 ± 59.09 s and post-CHL mean was 65.26 ± 49.68 s, indicating overlap in mean and SD. A one-way ANOVA between different days and sessions for speed was significantly different (*F* = 11.9, df = 13, *p* = 9.23E-26; Figure 3C). A paired *t*-test was not significant (*t*_239_ = 0.0259, *p* = 0.9794; Figure 3D). For time (sec), Cohen’s *d* was 0.0024 and effect size *r* was 0.0012, indicating a nonrelevant effect size. The direction of change was a decrease in time for CHL. For rotarod, the parameters speed (RPM) and time duration (sec) were not significantly different pre-CHL to post-CHL.

### 3.3. Balance beam

**Figure 4:**
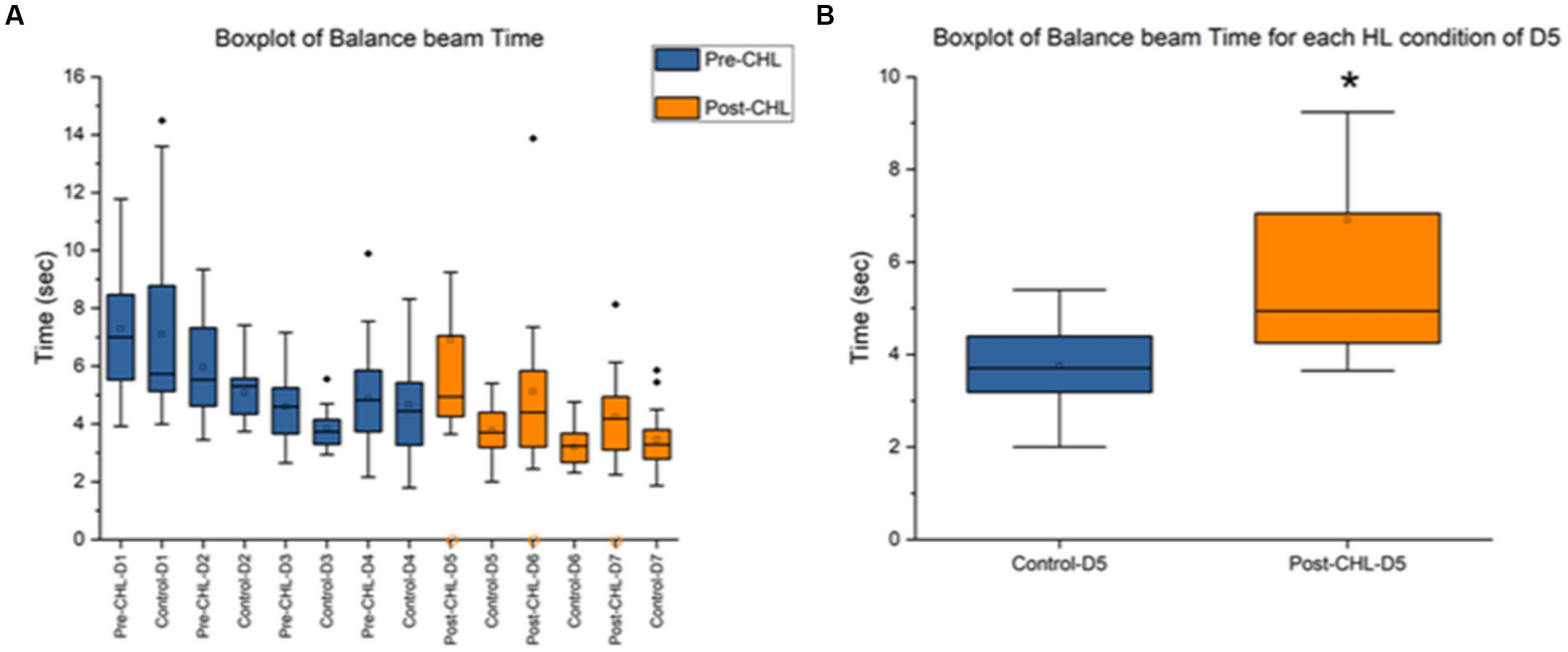
Balance beam pre-CHL and post-CHL for time to transverse (sec). Sample size of balance beam (*n* = 20). **(A)** Box and whisker plot of time to transverse (sec) datapoints across the days by group (control and pre/post-CHL). Pre-CHL is represented in blue and post-CHL is represented in orange. Some outliers are not plotted. Note the orange circles on the x-axis indicate rats with no movement on the balance beam where there were two rats with no movement in Post-CHL-Day 5, four rats in Post-CHL-Day 6, and four rats in Post-CHL-Day 7, respectively. As would be expected, prior to CHL when rats were equivalent, there was no difference. However, after day-5, the first day post-CHL, a significant difference is apparent. The following results were obtained for a repeated two-way ANOVA test for pre and post-CHL and different days: *F* = 6.23, df = 13, *p* = 4.14E-10. **(B)** Box and whisker plot of time to transverse (secs) datapoints session Day 5 for controls and post-CHL. Control is represented in blue and post-CHL is represented in orange. Note the dispersion between the groups. Control was significantly different than post-CHL. Some outliers are not plotted. On day 5, *p* < 0.05 was found. The asterisk * indicates significant *p* < 0.05.

Figure 4A displays the mean and range for time to transverse the balance beam. The day-5 control mean was 3.76 ± 0.89 s and post-CHL mean was 6.91 ± 6.05 s. A two-way ANOVA between pre and post-CHL groups and different days for balance beam was significantly different (*F* = 6.23, df = 13, *p* = 4.14E-10; Figure 4B). An unpaired t-test was significant (*t*_36_ = 2.2364, *p* = 0.0316) for time to transverse the balance beam, Cohen’s *d* was 0.7285 (medium effect size, near large effect size) and effect size *r* was 0.3422 (medium effect size). Day-5 post-CHL had two CHL rats with no movement. The day-6 control mean was 3.20 ± 0.63 s and post-CHL mean was 5.12 ± 2.68 s. An unpaired t-test was significant (*t*_34_ = 3.0012, *p* = 0.0050) for time to transverse the balance beam, Cohen’s *d* was 0.9863 (large effect size) and effect size *r* was 0.4423 (medium effect size). Day-6 post-CHL had four CHL rats with no movement. The day-7 control mean was 3.46 ± 0.97 s and post-CHL mean was 4.24 ± 1.51 s. An unpaired *t*-test was not significant (*t*_34_ = 1.8425, *p* = 0.0741), for time to transverse the balance beam, Cohen’s *d* was 0.6209 (medium effect size) and effect size *r* was 0.2965 (medium effect size, when rounded up). Day-7 post-CHL had four CHL rats with no movement. Only the CHL group had rats with no movement. There was no difference between control rats and rats pre-CHL during balance beam training. The direction of change for days post-CHL was an increase for CHL in time to transverse the balance beam.

### 3.4. EVM

**Figure 5:**
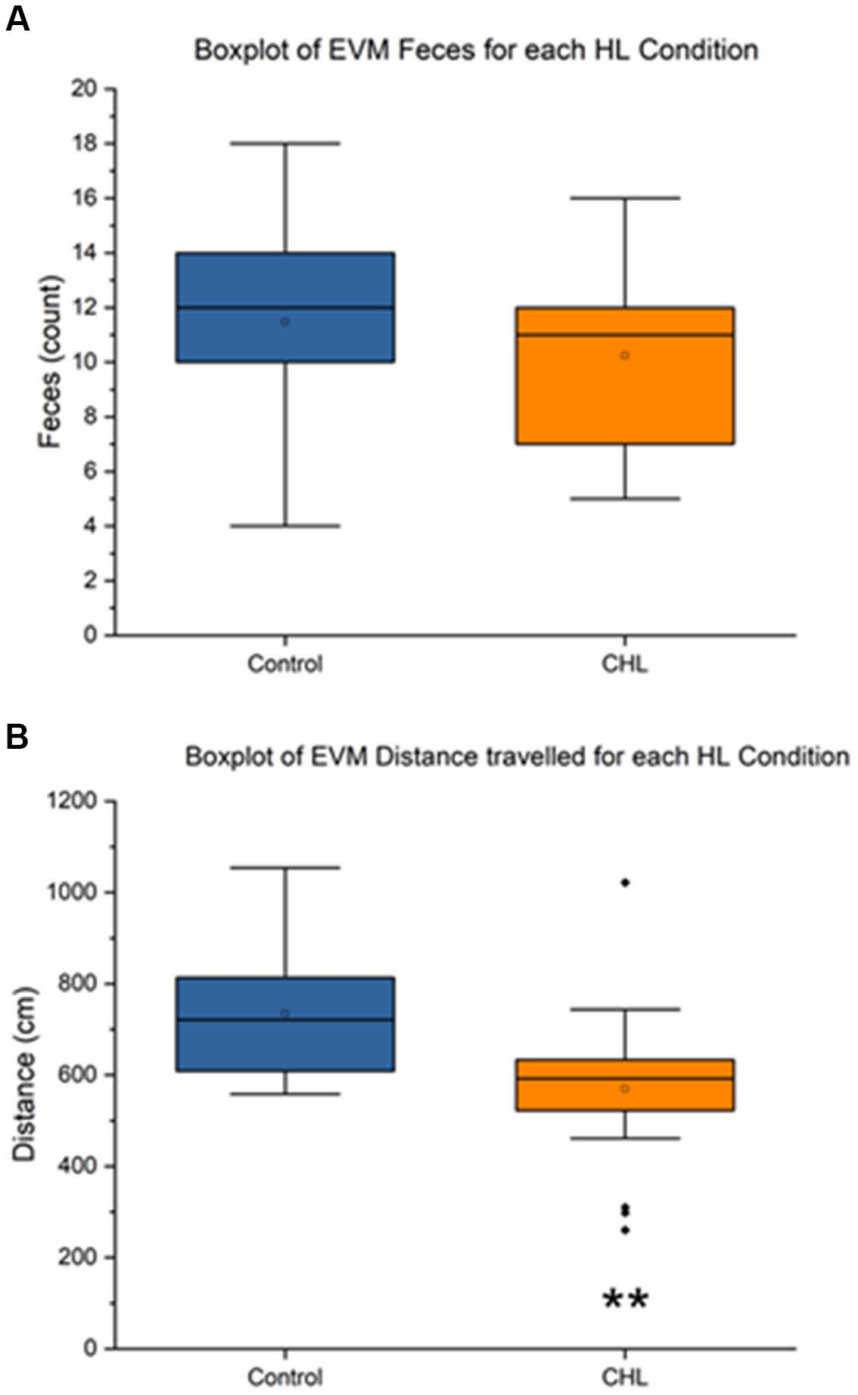
Elevator vertical motion control and CHL feces count, distance (cm), and speed (cm/s). Sample size of EVM (*n* = 34). For panels, control is represented in blue and CHL is represented in orange. **(A)** Box and whisker plot of feces count datapoints by group (control and CHL). Note that some rats in the CHL group did not have fecal boli. **(B)** Box and whisker plot of distance traveled (cm) datapoints by group (control and CHL). The asterisk ** indicates significant *p* < 0.01.

Figure 5 showed that the EVM feces control mean was 11.47 ± 3.957 and CHL mean was 10.24 ± 3.245 (Figure 5A). An unpaired t-test was not significant (*t*_32_ = 0.9655, *p* = 0.3416) for EVM feces, Cohen’s *d* was 0.3405 (small effect size) and effect size *r* was 0.1679 (large effect size). The direction of change was a slight decrease for CHL in feces during EVM. The EVM distance traveled control mean was 733.80 ± 133.18 cm and CHL mean was 569.72 ± 175.65 cm (Figure 5B). An unpaired t-test was significant (*t*_32_ = 2.9775, *p* = 0.0055) for EVM distance traveled, Cohen’s *d* was 1.0527 (large effect size) and effect size *r* was 0.4658 (medium effect size). The direction of change was a significant decrease for CHL in distance traveled after EVM.

### 3.5. FWR

**Figure 6:**
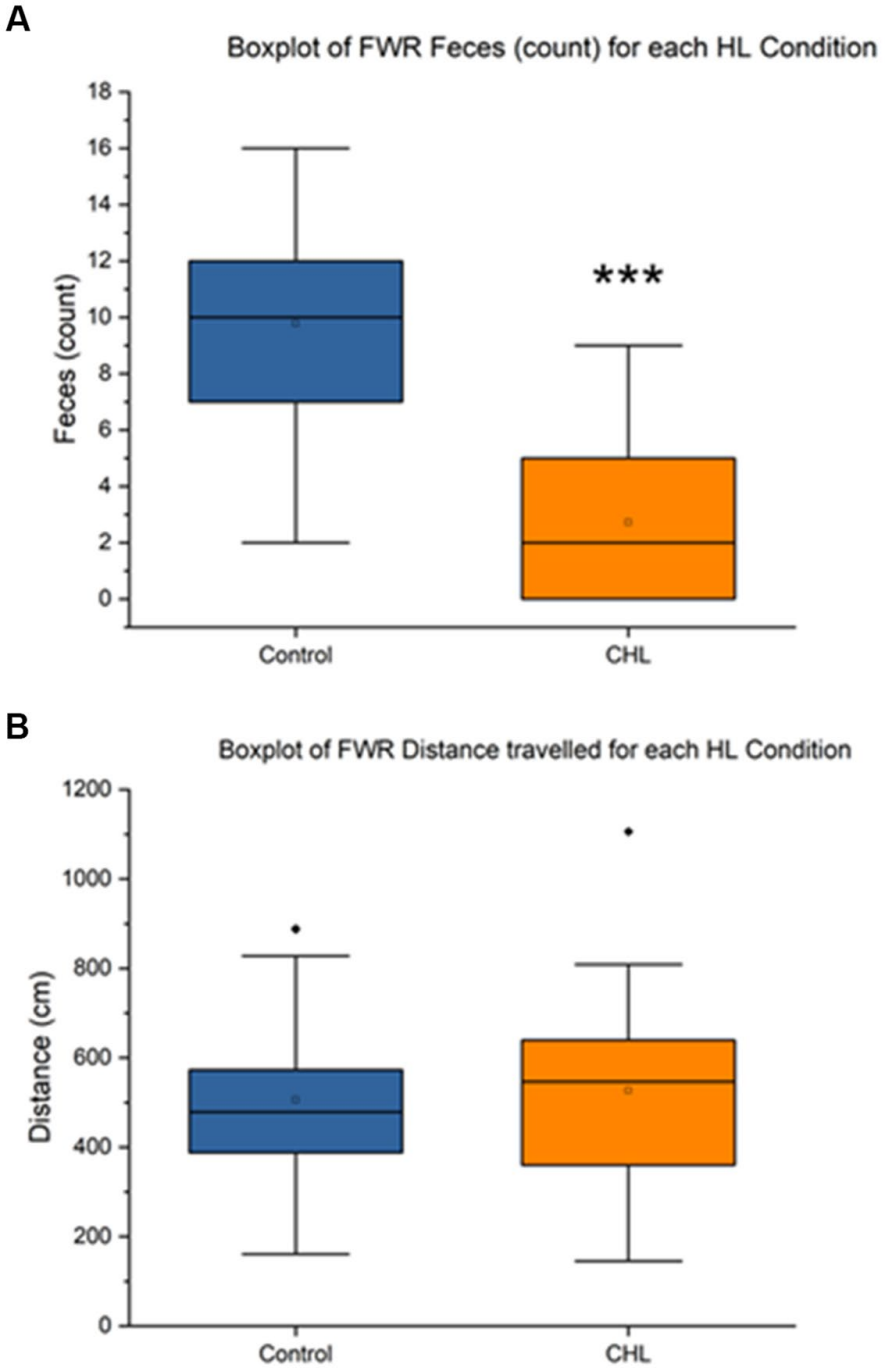
Ferris-wheel rotation control and CHL feces count, distance (cm), and speed (cm/s). Sample size of FWR (*n* = 36). For panels, control is represented in blue and CHL is represented in orange. **(A)** Box and whisker plot of feces count datapoints by group (control and CHL). *p* < 0.001 was found between groups. Note that some rats in the CHL group did not have fecal boli. **(B)** Box and whisker plot of distance traveled (cm) datapoints by group (control and CHL). The asterisk *** indicates significant *p* < 0.001.

Figure 6 showed that the FWR feces control mean was 9.78 ± 3.63 and CHL mean was 2.72 ± 2.78 (Figure 6A). An unpaired t-test was significant (*t*_34_ = 6.3589, *p* < 0.0001) for FWR feces, Cohen’s *d* was 2.1837 (very large effect size) and effect size *r* was 0.7374 (large effect size). The direction of change was a decrease for CHL in feces during FWR. The FWR distance traveled control mean was 505.84 ± 206.94 cm and CHL mean was 526.53 ± 236.74 cm (Figure 6B). An unpaired *t*-test was not significant (*t*_34_ = 0.2712, *p* = 0.7879) for FWR distance traveled, Cohen’s *d* was 0.0931 and effect size *r* was 0.0465. The direction of change was a non-significant increase for CHL in distance traveled after FWR.

## 4. Discussion

The present study had three main findings: (1) Rotarod was not significantly different between pre and post-CHL rats for time duration (sec) or speed (RPM), albeit the former nearly had a small effect size difference (Figure 3). (2) Balance beam time to transverse for day 5 and 6 was significantly different with a medium effect size between control and post-CHL rats (Figure 4). Time to transverse the balance beam was significantly greater for CHL rats. (3) The evaluation metric total distance traveled for EVM was significantly different between control and CHL rats (Figure 5). The evaluation metric defection number between control and post-CHL rats was significantly different with large effect sizes for FWR during motion stimulation (Figure 6). Rats with CHL defecated less. Less movement (EVM) may be associated with anxiety and alterations to defecation patterns (FWR) may result from autonomic disturbances due to the impact of hearing loss. Interestingly, for balance beam, the significant increase time to transverse due to CHL was abolished by day 3 post-surgery (non-significant, albeit medium effect size). Future studies should evaluate the neurological underpinnings of vestibular-related functioning in rats with CHL.

### 4.1. Vestibulomotor deficits may reflect anxiety—changes in balance and motion

Time to transverse the balance beam was significantly different between pre and post-CHL rats (medium effect size; Figure 4). Moreover, in the two significant days (Day-5 and Day-6 post-CHL) six rats were categorized as “no movements” and thus excluded from the dataset due to the unlimited time not terminating the task (10%, *t*_38_ = 1.4530, *p* = 0.1544 and 20%, *t*_38_ = 2.1794, *p* = 0.0356 of the group, respectively). On Day-7 there were four rats with no movement (i.e., excluded), although this day did not reach significance (20%, *t*_38_ = 2.1794, *p* = 0.0356 of the group). Rats with CHL not moving and taking longer to perform a previously learned task could be experiencing anxiety due to hearing loss (Antoine et al., 2017). In a complementary study, mice exposed to chronic low frequency noise (0.1 kHz) at 70 dB SPL experienced worse rotarod performance such as decreased retention time and balance beam impairments such as reduced crossings (Tamura et al., 2012). Interestingly, the balance beam has been used to study anxiety (Kalueff et al., 2005; Sweis et al., 2016). Here it has been shown that anxiety effects pathways involved in autonomic control and vestibulo-autonomic interactions through the parabrachial nucleus network, amygdaloid nucleus, infralimbic cortex, and hypothalamus (Balaban and Thayer, 2001). In the present study we do not claim to assess anxiety or confounding influences such as stress; therefore, these interactions may be up for debate. Future studies may want to assess the anxiogenic aspects of inducing hearing loss.

### 4.2. Autonomic effects—changes to defecation

Changes in defecation patterns observed after hearing loss could reflect alterations in the autonomic nervous system. In the rat, a pattern of dual, coordinated, parasympathetic innervation in the left colon regulates motor activity between the proximal colon and rectum, eliciting contractions in defecation (Tong et al., 2010). In rats, this is under control of the pontine defecation reflex center (Nagano et al., 2004). Although stress has been known to increase defecation (number and weight: Sanger et al., 2000), most of the studies are measured directly after the stress-inducer (Sanger et al., 2000; Suda et al., 2013), whereas our hearing loss model was evaluated 7 days post-CHL and resulted in less defection, in contrariety to published stressors. Therefore, perhaps generalized anxiety is a better description of the potential confounding influence in the CHL model, due to the long-term period elapsing between the surgery and the evaluation. The autonomic effects (changes to defecation seen in FWR, Figure 6) observed in rats with CHL could be considered epiphenomena related to anxiety. Nevertheless, the present experiments did not directly measure anxiety related metrics. In humans with hearing loss, a prime example is comorbidities of autonomic dysfunction accompanying the otologic symptoms (Pappas, 2003; Antoine et al., 2017; Yu and Li, 2018a,b). For example, in a study of humans with spontaneous vertigo having symptoms and findings consistent with poor autonomic regulation, patients who had tinnitus were relieved by a treatment strategy aimed to improve autonomic dysfunction (Pappas, 2003). The results in humans indicate a maladaptive outcome and/or compensatory change to the hearing loss insult (Merabet and Pascual-Leone, 2010). In neuroimaging, we recently found that hearing loss in humans not only affects auditory structures, but is brain-wide, multi-focal and impacts regions and tracts differently depending on auditory input and compensatory mechanisms (Manno et al., 2021b). Autonomic changes may reflect widespread autonomic tone differences associated with the sensory deficits due to hearing loss.

### 4.3. Comodulation of auditory and vestibular projections?

In the present study, we found CHL reduced distance traveled during open-field examination after EVM (vertical linear motion stimulation; Figure 5) and reduced defecation in FWR (centrifugal rotation stimulation; Figure 6). These assays stimulate similar and different vestibular functions through the vestibular portion of the vestibulocochlear nerve (Hitier et al., 2016) which contains projections from the semicircular canals, the otolith organs (saccule and utricle) and cochlea and then terminate on vestibular (inferior, lateral, medial and superior nuclei, and the related nucleus prepositus hypoglossi) and auditory nuclei (cochlear nuclear complex and the superior olivary complex) of the upper medulla (Altman and Bayer, 1980). We are uncertain if the vestibulomotor effects observed are due to comodulation (afferent or efferent signals from either auditory or vestibular dysregulation) in the vestibulocochlear nerve, further up in the brainstem or cortex (or related to anxiety). It is unknown whether cochlear and vestibular interaction occurs along the vestibulocochlear projection (Ryugo et al., 2011) to the medulla or higher-up in the cortex. The FWR assay stimulates the otolith organs by linear acceleration and the semicircular canals by angular acceleration (Curthoys, 2020). For elevator vertical motion (vertical plane: up-down) which predominately stimulates the saccule in rats, a decrease in distance traveled was observed (Figure 5). It is interesting to note the FWR did not produce changes in open field examination, but elicited reduced defecation. The opposite was true for EVM, producing changes in open field examination consisting of a decrease in the distance traveled, but no change to defecation. The unique difference between FWR and EVM is the former stimulates both otoliths and semicircular canals, while EVM only stimulates the saccule. Moreover, increases in open field behavior have been linked to stress (Roth and Katz, 1979) and anxiety or stress has been linked to open field defecation increases and ambulation decreases (Archer, 1973; Liebsch et al., 1998; Sestakova et al., 2013); thereby occupying both ends of the spectrum. We note defecation has a long histology in psychology research as a proxy for autonomic response (Hall, 1938, 1941; Bindra and Thompson, 1953; Lieblich and Guttman, 1968) and open field behavior as a measure of anxiety (Hall and Whiteman, 1951; Campbell and Candland, 1961; Snowdon et al., 1964; Ley, 1975; Roth and Katz, 1979). In regards to the current experiments, perhaps the 7-day period assessment after the CHL induction was too long post-surgery for the effects to be manifested. Rats for FWR reduced defecation and for EVM reduced distance traveled, possibly reflecting increased stress or anxiety (Hall, 1941; Archer, 1973). Future studies should attempt to dissect the otolith and semicircular canal contributions affecting balance and vestibular functioning in hearing loss and interaction of the vestibulocochlear projections.

### 4.4. Limitations, future directions, and conclusions

In the present manuscript, care was taken to follow identical procedures to our previous protocols to ensure replicability (Cai et al., 2007, 2010; Wang et al., 2012, 2015, 2017; Zhou et al., 2017; Qi et al., 2019; Manno et al., 2020). Despite this standardization effort, previous research has revealed the challenges with behavioral assessments (Crawley, 2007; Wahlsten, 2010). For example, we have previously demonstrated no gross inflammation and no pain response (Turner et al., 2019) with the CHL procedure (Manno et al., 2019), but we cannot exclude either of these contributing to the vestibulomotor effects observed. Further, rats could have experienced anxiety or general malaise for a long duration after the surgery, accounting for the alteration in locomotion. We cannot exclude the possibly that the results reflect anxiety, as we had no direct measure of anxiety in our vestibular phenotyping battery. Although we have previously performed sham experiments to account for the surgical effects (data not show -rats underwent anesthesia and sham surgery), neurobehavioral consequences of not hearing could cause significant stress or anxiety; therefore, confounding true CHL results with anxiogenic behavior not accounted for in our experimental design. Lastly, vertigo has been known to confound with hearing loss (Newman-Toker et al., 2016; Yu and Li, 2018b), which could account for behavioral results related to locomotion. Future investigations should explore directly altering the vestibular organs in temporary hearing loss (such as earplugging to reverse and establish a transient effect) in order to determine the interactions between the auditory and vestibular systems.

## Data availability statement

The datasets presented in this study can be found in online repositories. The names of the repository/repositories and accession number(s) can be found below: https://osf.io/whez9/ | https://francismanno.github.io/fmanno/ | https://doi.org/10.17605/OSF.IO/WHEZ9.

## Ethics statement

The animal study was reviewed and approved by the City University of Hong Kong.

## Author contributions

CL, YF, and FM organized the author contributions indicative of the CHL project at the City University of Hong Kong. FM, MK, YM, LP, VM, WC, ST, ZA, and MP performed the experiments. FM, PC, VB, ZA, MP, Y-LC, and CL analyzed the data. FM, YF, Y-LC, MP, and CL wrote the manuscript. MP, YF, Y-LC, MP, and CL financed the research. All authors contributed to the article and approved the submitted version.

## Funding

FM was a T32 NIH Postdoctoral Fellow grantee from the National Institute on Deafness and Other Communications Disorders (award no. 2T32DC000023-36A1). YF was supported by the General Program of National Natural Science Foundation of China (grant nos. 61671228, 61728107, and 81871349) and the Technology R&D Program of Guangdong (grant nos. 2017B090912006 and 2018B030333001). CL was supported by the Early Career Scheme, Research Grants Council of Hong Kong (project #21201217).

## Conflict of interest

The authors declare that the research was conducted in the absence of any commercial or financial relationships that could be construed as a potential conflict of interest.

## Publisher’s note

All claims expressed in this article are solely those of the authors and do not necessarily represent those of their affiliated organizations, or those of the publisher, the editors and the reviewers. Any product that may be evaluated in this article, or claim that may be made by its manufacturer, is not guaranteed or endorsed by the publisher.
